# On the Determination of Uncertainty and Limit of Detection in Label-Free Biosensors

**DOI:** 10.3390/s18072038

**Published:** 2018-06-26

**Authors:** Álvaro Lavín, Jesús de Vicente, Miguel Holgado, María F. Laguna, Rafael Casquel, Beatriz Santamaría, María Victoria Maigler, Ana L. Hernández, Yolanda Ramírez

**Affiliations:** 1 Centre for Biomedical Technology, Optics, Photonics and Biophotonics Laboratory, Campus Montegancedo, Universidad Politécnica de Madrid, 28223 Madrid, Spain; alvaro.lavin@upm.es (A.L.); mariafe.laguna@upm.es (M.F.L.); rafael.casquel@upm.es (R.C.); beatriz.santamaria@ctb.upm.es (B.S.); m.maigler@biod.es (M.V.M.); ana.lopez@upm.es (A.L.H.); y.ramirez@biod.es (Y.R.); 2 Department of Applied Physics, Escuela Técnica Superior de Ingenieros Industriales, Universidad Politécnica de Madrid, C/José Gutiérrez Abascal, 2, 28006 Madrid, Spain; jvo@etsii.upm.es; 3 Laboratorio de Metrología y Metrotecnia, Escuela Técnica Superior de Ingenieros Industriales, Universidad Politécnica de Madrid, C/ José Gutiérrez Abascal, 2, 28006 Madrid, Spain; 4 BioOptical Detection, Centro de Tecnología Biomédica, Campus Montegancedo, 28223 Madrid, Spain

**Keywords:** label-free biosensing performance, calculation of the limit of detection, limit of quantification, measurement uncertainty

## Abstract

A significant amount of noteworthy articles reviewing different label-free biosensors are being published in the last years. Most of the times, the comparison among the different biosensors is limited by the procedure used of calculating the limit of detection and the measurement uncertainty. This article clarifies and establishes a simple procedure to determine the calibration function and the uncertainty of the concentration measured at any point of the measuring interval of a generic label-free biosensor. The value of the limit of detection arises naturally from this model as the limit at which uncertainty tends when the concentration tends to zero. The need to provide additional information, such as the measurement interval and its linearity, among others, on the analytical systems and biosensor in addition to the detection limit is pointed out. Finally, the model is applied to curves that are typically obtained in immunoassays and a discussion is made on the application validity of the model and its limitations.

## 1. Introduction and Review of Basic Concepts

Label-free biosensing is an exciting field in which promising and new biosensing concepts and devices are being developed by scientific community. Some examples of published review articles [1,2,3,4] show the multiple types of biosensors and their corresponding performance reported. Although the calculation of the measurement uncertainty and the Limit of Detection (LoD) may be considered a surpassed subject and a worthless matter of study, there is still a controversy about the way in which these figures are calculated in the majority of the articles. For example, authors sometimes report the uncertainty with a single standard deviation (*σ*), or estimate the LoD by the conventional 3*σ* without taking into account the reader resolution and sometimes only the resolution of the reader instrument is borne in mind to estimate the LoD.

On the other hand, in the field of research and development of pharmaceutical articles, the validation of an analytical procedure is the process by which it is established, by laboratory studies, that the performance characteristics of the procedure meet the requirements for the intended analytical application [5,6]. Typical validation characteristics used in method validation are accuracy, precision, specificity, detection limit, quantification limit linearity, measuring interval and robustness. Different organizations, like Eurachem, United States Pharmacopeia (USP) and International Conference on Harmonization (ICH) have defined these characteristics, in order to harmonize the regulations in USA, Japan and the European Union [5,6,7].

In the field of research into new biosensing devices, the aforementioned characteristics affect the definition of the performance, not only for a reliable comparison among the different biosensors, but also for the development of new prototypes. This is even more important if we want to make the leap to their commercialization as products, such as for the measurement of a given biomarkers of human disease.

The ability to detect small amounts of molecules dissolved in a solution using any analytical method or through some type of biosensor can be quantified through the limit of detection. The limit of detection is expressed in units of concentration and, following IUPAC definition, indicates the smallest solute concentration than a given analytical system is able to distinguish with a reasonable reliability of a sample without analyte [8]. Alternative, USP’s definition introduces the limit of detection as the lowest amount of analyte in a sample that can be detected, but not necessarily quantified, under the stated experimental conditions [5]. This definition immediately leads to the concept of quantification limit, which is defined as the minimum amount of analyte in a sample whose concentration can be determined with acceptable accuracy and precision under certain experimental conditions. Procedural definitions based on statistical parameters such as the standard deviation and the slope of the calibration curve; and the probabilities of false positives or negatives associated with a critical value have been made by different organizations [6,8,9,10]. This article seeks to delve into these concepts. In the scientific literature, several works can be found that deepen and clarify the concept of limit of detection, affecting in some cases specific sensors [11,12,13,14]. A classic work like [15] discusses the degree of confusions surrounding the use of LoD and reasons why sometimes estimations of LoD are unrealistically low. It is also necessary to mention here the excellent recent works in which the different methods of obtaining the limit of detection for chemical sensors are listed [11,16,17].

**Figure 1:**
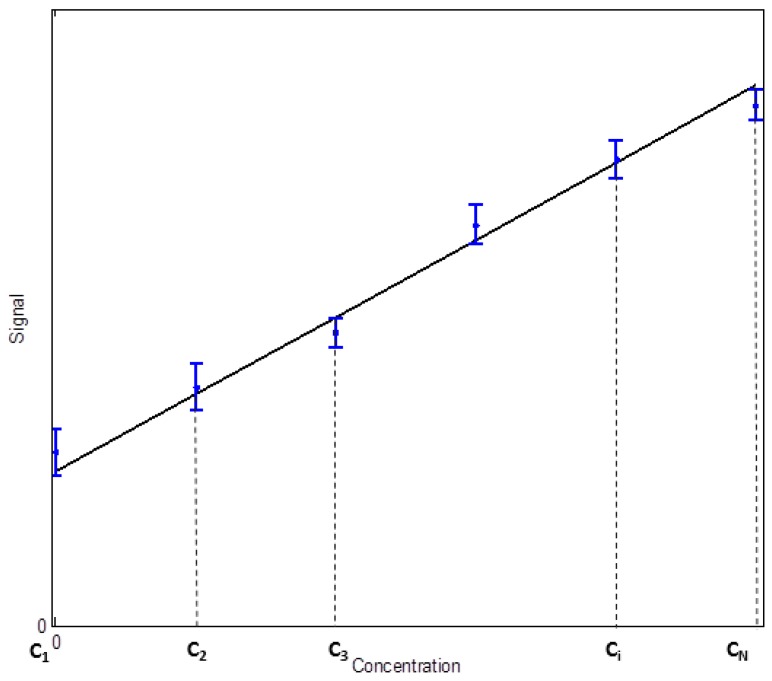
Calibration function.

The LoD is commonly used as evidence of the quality of a biosensor. It is usually obtained indirectly through a linear calibration function constructed from a linear regression performed on a set of measurements of instrument response versus concentration. Through this simple model, additional information about accuracy, precision, quantification limit, linearity and measuring interval can be obtained. Figure 1 depicts an idealized a calibration function based on *N* calibration points over a range of concentrations from zero concentration *C_1_* to concentration *C_N_*. *n* independent measurements are repeated in each concentration of the calibration function. From these measurements, we can obtain the mean values and standard deviations of the signal at each concentration point and finally, through the linear regression, the parameters *a* and *b* of the linear calibration function, where *a* is the slope of the straight line and *b* the intersection with the vertical axis:

(1) y=a C+b

In most label-free biosensors the sensing surface has a limited extent, so that the sensing curve that we obtain in an immunoassay tends to saturation. We usually use the lower concentration range to define our calibration curve, in which we assume that the sensitivity is quasi-constant and the sensor measures under optimal conditions [10]. To adjust the complete sensing curve, including the saturation interval or ‘plateau’, other fits are used such as sigmoidal curve. In general, it can be stated that as we move away from low concentrations, the sensitivity decreases and the uncertainty of the concentration increases, tending to infinity in the plateau, which makes this interval not useful as a calibration curve in the sense we have indicated. Nevertheless, it may be interesting to make other adjustments using the entire calibration curve to obtain other experimental parameters (see, for example [18]) such as the dynamic signal range [8] or the working range [19].

The slope (*a*) of the function is the analytical sensitivity, that is the change in the response of an analytical system or biosensor for a small variation in the analyte concentration [6,8]. After determining the calibration curve, a biosensing signal (*y*) of the biosensor may be associated with a concentration (*C*) of an analyte in the solution. The uncertainty in the determination of *C* will depend, among other factors, of the uncertainty with which we determine each of the points of the calibration curve and the possible nonlinearity of the data from which it is built. From now on, we will assume that the nonlinearity of the data is negligible compared to the uncertainty due to lack of repeatability in measurements. It is recommended, for the establishment of linearity in a range, a minimum of five concentrations [5].

The uncertainty of our signal can only be determined if a sufficient number of observations are repeated. It is customary to assume as a hypothesis that all these measurements follow a Gaussian (Equation (2)) around a central value *μ*. In a Gaussian distribution function, *σ* parameter is the standard deviation:

(2) $$f_{G}=\frac{1}{\sigma \sqrt{2\pi }}e^{-\frac{1}{2}{\left(\frac{y-\mu }{\sigma }\right)}^{2}}$$

**Figure 2:**
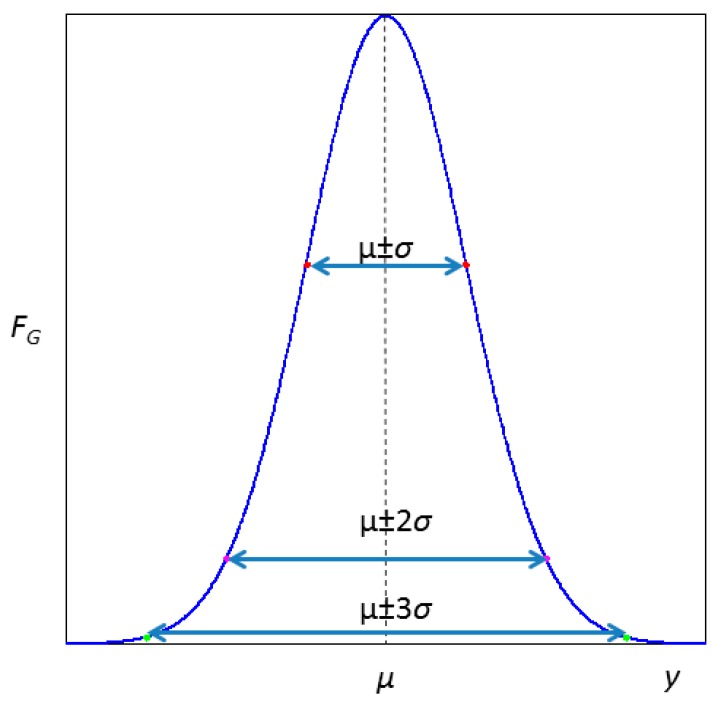
Gaussian function.

In Figure 2 this mathematical function is represented and its relationship with *μ* and *σ* parameters. From the Gaussian function, by integration into a range, the probability of finding the variable in it is obtained. If a set of measurements fit Gaussian statistical model, almost 32% of them will be outside the interval [*μ − σ*, *μ + σ*], about 5% will be outside the interval [*μ −* 2*σ*, *μ +* 2*σ*] and less than 0.3% will be outside the interval [*μ −* 3*σ*, *μ +* 3*σ*].

When a set of *n_i_* measurements is carried out in the ith point of a calibration curve, we can compute estimations for *μ* and *σ* with the following expressions:

(3) $${\bar{y}}_{i}=\frac{\sum_{j=1}^{n_{i}}y_{ij}}{n_{i}}$$

(4) $$\;s_{i}=\sqrt{\frac{\sum_{j=1}^{n_{i}}{\left(y_{ij}-{\bar{y}}_{i}\right)}^{2}}{n_{i}-1}}$$

The LoD of an analytical system can be estimated by reiterating $n_{B}$ measurements of the signal on a sample with null concentration or blank. If the fluctuations of the measured values follow a normal distribution, we can estimate the mean and standard deviation of the blank signal $y_{B}$ from Equations (3) and (4). Up to this point, only the statistical uncertainty is bearing in mind, considering negligible other sources of uncertainty, as we will explain later in this article.

**Figure 3:**
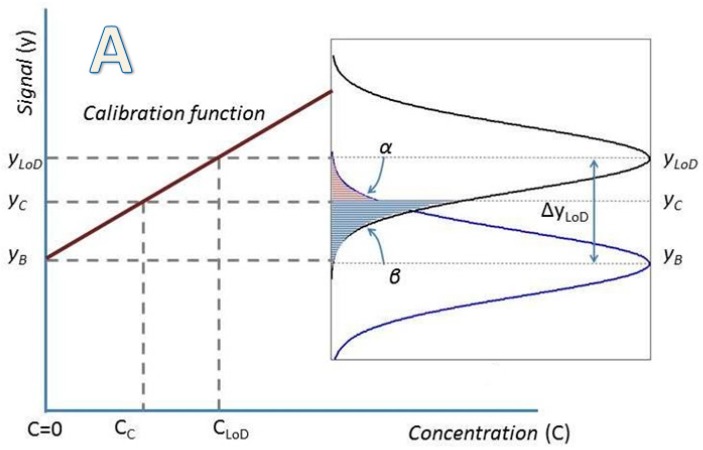

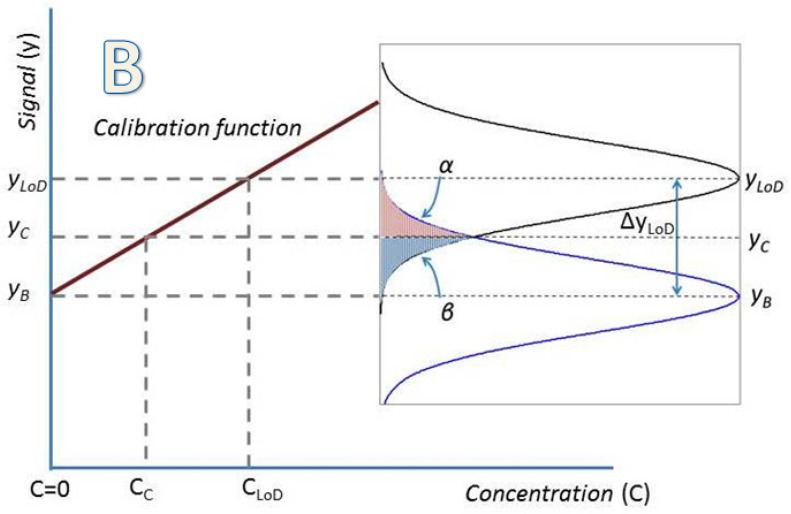
(**A**) Critical value, Limit of Detection, false positives and false negatives for the general case; (**B**) Critical value, Limit of Detection, false positives and false negatives for the case α = β.

LoD is strongly related in origin with the concept of critical value. The critical value (*y_C_*) of the signal *y*, is defined as the value, the exceeding of which leads, for a given error probability *α*, to the decision that the concentration is not zero [6,9,10,20] when measuring a measurand without analyte. The detection limit of the signal *y* (*y_LoD_*) is defined as the central value of a Gaussian distribution which probability of being below critical value *y_C_* is *β*. Error probabilities *α* and *β* are, respectively, the probabilities of false positive and false negative, and should be chosen according to the confidence level required. Figure 3 shows graphically the relations between these parameters for cases α≠β and α=β.

For a better understanding of the relations between *y_B_*, *y_C_*, *y_LoD_*, *α* and *β* we indicate the following examples:

A.—If we choose *α* = *β* = 0.05 (5%): then *y_LoD_ − y_C_* = 1.645 σ, *y_C_ − y_B_* = 1.645 σ and *y_LoD_ − y_B_* = 3.29 σ

B.—If we maintain *α* = *β* fixing *y_LoD_ − y_B_* = 3σ then *α* = *β* = 0.067 (6.7%). k=3 is the value recommended by [15].

C.—If we choose *y_LoD_ − y_B_* = 3σ and *y_LoD_* = *y_C_*, then the error probability *α* would be extremely small, less than 0.0015 (0.15%), but error probability *β* would be as great as 0.5 (50%).

IUPAC establishes the following relation between *y_B_* and *y_LoD_* ([8], page 839):

(5) $$y_{LoD}=y_{B}+ks_{B}$$

where *y_B_* is the mean of the blank measurements, *s_B_* is the standard deviation of the blank measurements. The numerical factor *k* is chosen according to the confidence level desired. (See Figure 3 and its subsequent explanation).

The concentration at the LoD (*C_LoD_*), normally noted as LoD can be easily calculated from the analytical sensitivity *a* of a previously done calibration function or calibration curve:

(6) $$C_{LoD}=\frac{y_{LoD}-y_{B}}{a}=\frac{ks_{B}}{a}$$

Many articles use this convention to estimate the LoD of a given biosensing system. This equation, in appearance, only takes into account the statistical uncertainty considering other sources insignificant, for example the readout instrument resolution. Moreover, different influencing factors that affect the measurement, such as temperature or relative humidity, uncertainty in concentrations used during calibration process or day-to-day fluctuations, could add uncertainty and worsen the value of the limit of detection [21]. Therefore, we believe that in the definition of the limit of detection of Equation (6) the meaning of $s_{B}$ should be clarified beyond the pure statistical definition of the standard deviation. We can avoid the measurement of the magnitudes associated with different influencing factors by making our instruments, commercial or laboratory, measure in certain reference conditions; or we can neglect, under certain hypothesis, other contributions to uncertainty, but, for example, it is important to consider that the readout resolution of the measuring instruments have to be taken into account for calculating the limit of detection [16]. When $s_{B}$ is small in comparison with the readout instrument resolution R ($s_{B}<0.3R$), the standard deviation cannot be calculated by using frequency-based statistics (type A estimation) and a different approach should be applied [22]. When R is high enough, $s_{B}$ becomes zero (all repeated measurement are equal, the instrument shows perfect repeatability) and the LoD would be zero too if only $s_{B}$ is taking into account. An alternative could be using ${\left(s_{B}^{2}+R^{2}/12\right)}^{1/2}$ instead of simply $s_{B}$ (see [23], rule 1) as we propose in this work.

## 2. Determination of Uncertainty in a Measuring Interval and the Limit of Detection through the Calibration Function Data

**Figure 4:**
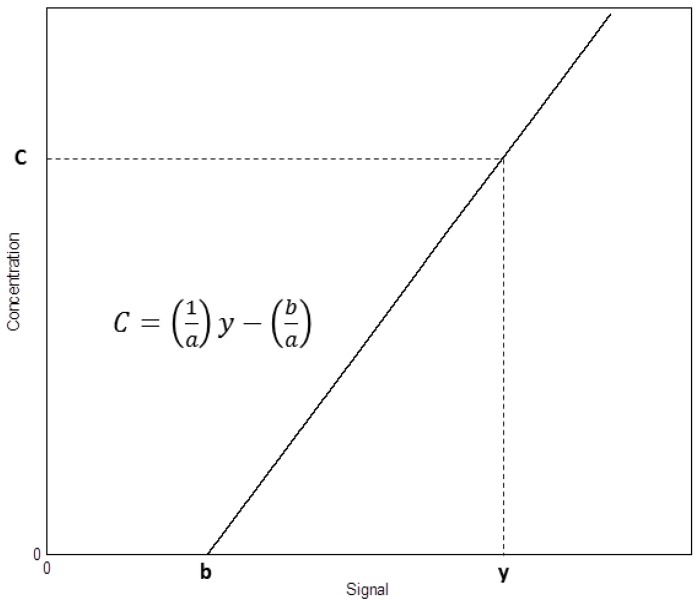
Concentration *C* versus response *y* through the calibration function.

In the following, we will delve into the process of obtaining the calibration curve, in how we attribute uncertainty to the concentration measurements and in the relationship between the uncertainty in the measurement of the concentration and the limit of detection. Once the calibration curve is determined, it translates the sensor signal into analyte concentration for future measurements in similar conditions (concentration, analyte). The reversal of the axes (inverse calibration function), representing the signal on the abscissa axis and the concentration on the ordinates, reflects this situation better (Figure 4). Since we want to obtain the uncertainty in the concentration, it would be represented as a vertical bar.

When we use the calibration curve (Equation (1)) to determine a concentration we use the following expression (inverse calibration function):

(7) $$C=\frac{y-b}{a}$$

If the parameters *a* and *b* have an uncertainty due to the process of determination of the calibration curve, that uncertainty influences the uncertainty of *C*. Among other methods [6], we can use the law of propagation of uncertainties for correlated input quantities to determine the standard uncertainty in the concentration [21,24]:

(8) $$u_{C}^{2}={\left(\frac{\partial C}{\partial y}\right)}^{2}u_{y}^{2}+{\left(\frac{\partial C}{\partial b}\right)}^{2}u_{b}^{2}+{\left(\frac{\partial C}{\partial a}\right)}^{2}u_{a}^{2}+2\left(\frac{\partial C}{\partial a}\right)\left(\frac{\partial C}{\partial b}\right)u_{a}u_{b}r\left(a,b\right)$$

In Equation (8): *u_C_* is the combined standard uncertainty for concentration that is estimated combining the standard uncertainties *u_y_*, *u_b_* and *u_a_*; we suppose *a* and *b* are the only correlated quantities; and *r(a,b)* is the estimated correlation coefficient for *a* and *b* quantities [21,24].Applying Equation (8) to Equation (7) we obtain:

(9) $$u_{C}^{2}={\left(\frac{1}{a}\right)}^{2}u_{y}^{2}+{\left(\frac{-1}{a}\right)}^{2}u_{b}^{2}+{\left(\frac{b-y}{a^{2}}\right)}^{2}u_{a}^{2}+2\left(\frac{y-b}{a^{3}}\right)u_{a}u_{b}r\left(a,b\right)$$

In Equation (9) parameters *a*, *b*, *u_a_*, *u_b_* and *r(a,b)* are obtained from a set of *n_i_* signal measurements *y_ij_* {j = 1,.. n_i_} taken for N concentrations C*_i_* {*i* = 1,…N}. Assuming that signal measurements *y_j_*, for each concentration, have a normal distribution, they will be characterized by its means and standard deviations {$y_{i},s_{y_{i}}$} (See Equations (3) and (4)). Additionally, if we assume negligible the uncertainty in the independent variable *C_i_* compared to the uncertainty of the signal *y_i_* (Equation (10)), we can obtain the parameters *a* and *b* from the N triads $\left\{C_{i},y_{i},s_{y_{i}}\right\}$ and Equations (11)–(13) [25]:

(10) $$\;s_{y_{i}}\gg as_{C_{i}}$$

(11) $$a=\frac{1}{\Delta }\left|\begin{array}{cc} \sum_{i=1}^{N}\frac{1}{s_{y_{i}}^{2}} & \sum_{i=1}^{N}\frac{y_{i}}{s_{y_{i}}^{2}}\\ \sum_{i=1}^{N}\frac{C_{i}}{s_{y_{i}}^{2}} & \sum_{i=1}^{N}\frac{C_{i}y_{i}}{s_{y_{i}}^{2}} \end{array}\right|$$

(12) $$\;b=\frac{1}{\Delta }\left|\begin{array}{cc} \sum_{i=1}^{N}\frac{y_{i}}{s_{y_{i}}^{2}} & \sum_{i=1}^{N}\frac{C_{i}}{s_{y_{i}}^{2}}\\ \sum_{i=1}^{N}\frac{C_{i}y_{i}}{s_{y_{i}}^{2}} & \sum_{i=1}^{N}\frac{C_{i}^{2}}{s_{y_{i}}^{2}} \end{array}\right|$$

(13) $$\Delta =\left|\begin{array}{cc} \sum_{i=1}^{N}\frac{1}{s_{y_{i}}^{2}} & \sum_{i=1}^{N}\frac{C_{i}}{s_{y_{i}}^{2}}\\ \sum_{i=1}^{N}\frac{C_{i}}{s_{y_{i}}^{2}} & \sum_{i=1}^{N}\frac{C_{i}^{2}}{s_{y_{i}}^{2}} \end{array}\right|$$

Assuming homoscedasticity, $s_{y_{i}}$ similar for every calibration point, $s_{y}=s_{y_{i}}$, Equations (11)–(13) become:

(14) $$\Delta \prime =\frac{1}{s_{y}^{4}}\left(N\sum_{i=1}^{N}C_{i}^{2}-{\left(\sum_{i=1}^{N}C_{i}\right)}^{2}\right)$$

(15) $$\;a=\frac{N\sum_{i=1}^{N}C_{i}y_{i}-\left(\sum_{i=1}^{N}C_{i}\right)\left(\sum_{i=1}^{N}y_{i}\right)}{N\sum_{i=1}^{N}C_{i}^{2}-{\left(\sum_{i=1}^{N}C_{i}\right)}^{2}}$$

(16) $$\;b=\frac{\left(\sum_{i=1}^{N}y_{i}\right)\left(\sum_{i=1}^{N}C_{i}^{2}\right)-\left(\sum_{i=1}^{N}C_{i}y_{i}\right)\left(\sum_{i=1}^{N}C_{i}\right)}{N\sum_{i=1}^{N}C_{i}^{2}-{\left(\sum_{i=1}^{N}C_{i}\right)}^{2}}$$

The process of least squares fitting used for obtaining the parameters *a* and *b* of the calibration function implies the solution of the following overdetermined system of equations:

(17) Ax=y

where:

(18) $$\begin{array}{ccc} A=\left[\begin{array}{c} \begin{array}{cc} C_{1} & 1\\ C_{2} & 1\\ \cdot & \cdot \end{array}\\ \begin{array}{cc} \cdot & \cdot \\ C_{N} & 1 \end{array} \end{array}\right] & x=\left[\begin{array}{c} a\\ b \end{array}\right] & y=\left[\begin{array}{c} \begin{array}{c} y_{1}\\ y_{2}\\ \cdot \end{array}\\ \begin{array}{c} \cdot \\ y_{N} \end{array} \end{array}\right] \end{array}$$

Solution of Equation (17) is:

(19) $$x={\left(A^{T}A\right)}^{-1}\cdot \left(A^{T}y\right)=My$$

where:

(20) $$M={\left(A^{T}A\right)}^{-1}\cdot A^{T}$$

According to the evaluation of measurement data—Supplement 2 to the “Guide to the expression of uncertainty in measurement”—Extension to any number of output quantities [26] covariance matrix of the fitting parameters *a* and *b* could be obtained through the following expression:

(21) $$\;\mathrm{cov}\left(x\right)=M\mathrm{cov}\left(y\right)M^{T}$$

The hypothesis of homoscedasticity allows expressing the covariance matrix of *y* as $\mathrm{cov}\left(y\right)=u_{y}^{2}I$ where ***I*** is the identity N × N matrix, and Equation (21) could be expressed as:

(22) $$\;\mathrm{cov}\left(x\right)=\left[\begin{array}{cc} u_{a}^{2} & u_{ab}\\ u_{ab} & u_{b}^{2} \end{array}\right]=\frac{s_{y}^{2}}{N\sum_{i=1}^{N}C_{i}^{2}-{\left(\sum_{i=1}^{N}C_{i}\right)}^{2}}\left[\begin{array}{cc} N & -\sum_{i=1}^{N}C_{i}\\ -\sum_{i=1}^{N}C_{i} & \sum_{i=1}^{N}C_{i}^{2} \end{array}\right]$$

Thus:

(23) $$u_{a}=s_{y}\sqrt{\frac{N}{N\sum_{i=1}^{N}C_{i}^{2}-{\left(\sum_{i=1}^{N}C_{i}\right)}^{2}}}$$

(24) $$\;u_{b}=s_{y}\sqrt{\frac{\sum_{i=1}^{N}C_{i}^{2}}{N\sum_{i=1}^{N}C_{i}^{2}-{\left(\sum_{i=1}^{N}C_{i}\right)}^{2}}}$$

(25) $$u_{ab}=s_{y}^{2}\frac{-\sum_{i=1}^{N}C_{i}}{N\sum_{i=1}^{N}C_{i}^{2}-{\left(\sum_{i=1}^{N}C_{i}\right)}^{2}}$$

Finally, correlation coefficient r(a,b) can be obtained through relation $u_{ab}=u_{a}u_{b}r\left(a,b\right)$ [21,24]:

(26) $$r\left(a,b\right)=\frac{-\sum_{i=1}^{N}C_{i}}{\sqrt{N\sum_{i=1}^{N}C_{i}^{2}}}$$

The expanded uncertainty $U_{C}$ is obtained from Equation (9) by multiplying the combined standard uncertainty *u_C_* by a coverage factor *k* and defines an interval that should include a large fraction of the distribution of values that could reasonably be attributed to the concentration [21,24]. The value of *k* considered is 3 to have a level of confidence greater than 99%:

(27) $$\;U_{C}=k\left[\frac{1}{a}\sqrt{u_{y}^{2}+u_{b}^{2}+{\left(\frac{b-y}{a}\right)}^{2}u_{a}^{2}+2\left(\frac{y-b}{a}\right)u_{a}u_{b}r\left(a,b\right)}\right]$$

The above expression allows the calculation of the uncertainty in the value of the concentration *C* obtained for a response *y*. When the concentration approximates to zero, the signal *y* tends to *b* and Equation (27) becomes:

(28) $$\;U_{y\to b}=k\left[\frac{1}{a}\sqrt{u_{B}^{2}+u_{b}^{2}}\right]$$

In Equation (28) the subscript *B* refers to the zero concentration or *blank* and *u_B_* to the uncertainty of the signal for the zero concentration. If we assume that *b* is determined without uncertainty. Equation (28) becomes similar to the definition of IUPAC expressed in Equation (6). The difference is that the statistical estimator *s_B_* has been replaced by the standard uncertainty associated with the null concentration:

(29) $$\;C_{LoD}=\frac{ks_{B}}{a}=U_{y\to b}=\frac{ku_{B}}{a}$$

Considering this change in Equation (29), different sources of uncertainty can be analyzed. At least is recommend to analyze two of them: the statistical uncertainty mainly due to the lack of repeatability (*u_s_*) and the resolution (*u_R_*). As indicated at the end of the previous section, there are other contributions whose analysis would lead us to more complex and less general models. For the statistical uncertainty is possible to use as the best estimator of the data dispersion, the standard deviation of the mean, which is obtained by dividing by $\sqrt{n}$ the standard deviation *s_B_* of the sample [24]:

(30) $$u_{s}=\frac{S_{B}}{\sqrt{n}}$$

When the resolution of the readout system is a significant source of uncertainty comparable to the statistical one, especially when $s_{B}<0.3R$ [22], it is required to consider this uncertainty associated with the resolution *R* of the instrument. Thus, *u_R_* can be calculated by the Equation (31), assuming a uniform distribution of probability between −R/2 and R/2 [21,24]:.

(31) $$u_{R}=\frac{R}{\sqrt{12}}$$

Once $s_{B}$ is replaced by ${\left(s_{B}^{2}+R^{2}/12\right)}^{1/2}$ (see [23], rule 1) taking into account the contribution of the resolution R of the instrument, Equation (28) could be rewritten as:

(32) $$C_{LoD}=k\left[\frac{1}{a}\sqrt{\frac{s_{B}^{2}}{n}+\frac{R^{2}}{12}+u_{b}^{2}}\right]$$

Both factors: Statistical uncertainty (*u_s_*) and resolution (*u_R_*), are taken into account in the calculation of the *C_LoD_* of Equation (32). However, either *u_R_*, *u_s_* or *u_b_* could be negligible depending on the biosensing system analyzed.

Keeping the same criteria Equation (27) becomes:

(33) $$U_{C}=k\left[\frac{1}{a}\sqrt{\frac{s_{y}^{2}}{n}+\frac{R^{2}}{12}+u_{b}^{2}+{\left(\frac{b-y}{a}\right)}^{2}u_{a}^{2}+2\left(\frac{y-b}{a}\right)u_{a}u_{b}r\left(a,b\right)}\right]$$

Equation (33) gives us an uncertainty band that characterizes each point of our measurement range.

In the case that the sensor studied derives in a commercial design since the final user makes a single measurement, Equation (33) with *n* = 1 can give us an estimate of the uncertainty of that instrument. In that case, $s_{y}^{2}$ could be replaced by the mean or the maximum of the standard deviations of the calibration curve for the heteroscedastic case. In the same way, the terms ${\left(\frac{b-y}{a}\right)}^{2}u_{a}^{2}$ and $2\left(\frac{y-b}{a}\right)u_{a}u_{b}r\left(a,b\right)$ could be replaced by its maximum value in the measuring interval in order to attribute to *U* a single numerical value.

## 3. Analysis of Standard Immunoassay Situations

In Section 3.1, we apply the model to artificially generated data with the fundamental characteristics of the typical curves obtained in immunoassays. This study helps us analyze the validity and limitations of the linear model as a generator of reliable calibration curves. In Section 3.2, a real experimental case is analyzed, studying the best options for the calibration curve.

### 3.1. Simulated Immunoassay

**Figure 5:**
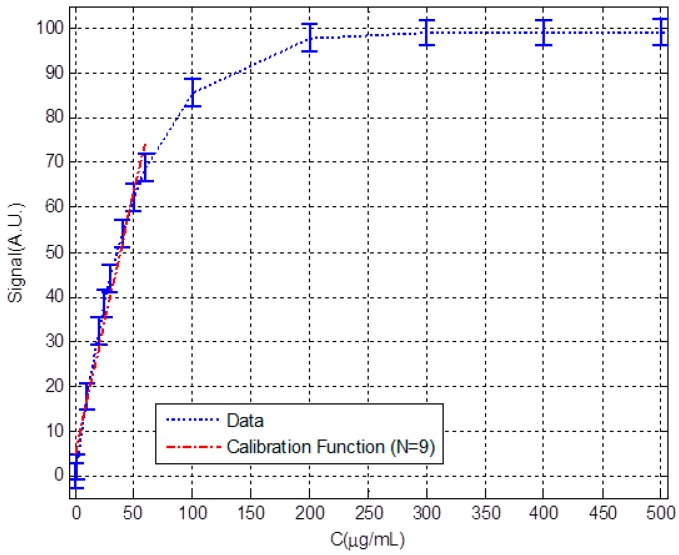
Theoretical sets of points representing an immunoassay and calibration function build for the first nine points.

To analyze and discuss the validity of the model and its limitations we will apply it to a set of regular data generated from a theoretical immunoassay model (Figure 5). The horizontal axis represents a concentration between 0 and 500 μg/mL. In the vertical axis, to generalize, a transduction signal in arbitrary units is represented that reaches up to 100 in the saturation. We assume the number of repetitions of the signal measurement at each point *n* = 5, a uniform standard deviation for each measurement point of 3 arbitrary units (A.U.) and a resolution of 3 A.U. Finally, in all calculations, *k* = 3 is used as the uncertainty constant or coverage factor. Figure 5 represents these data and the calibration function built for the set of the 9 first points. Numerical values are listed in Table 1.

**Figure 6:**
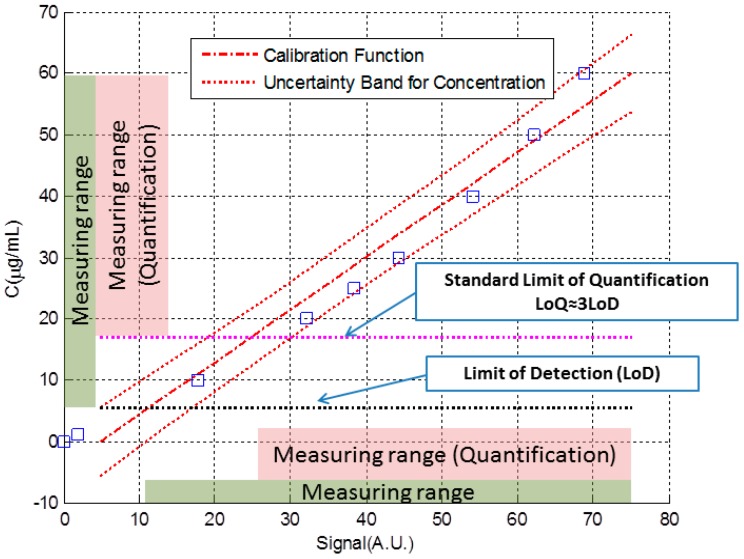
Data, calibration line, uncertainty band, limits of detection and quantification and measuring intervals for the given example.

Figure 6 shows in more detail the interval at which linear regression was performed. In this case, the axes have been exchanged: the signal is now represented on the horizontal axis and the corresponding concentration is represented on the vertical axis. This is the natural way in which the calibration curve translates the signal into a concentration value. In Figure 6 we can see the data, the calibration line obtained from Equations (7), (15) and (16), the uncertainty band obtained from Equation (33) and the limits of detection and quantification, obtained from Equation (32) and the standard definition of the quantification limit.

It can be seen that the uncertainty band is narrower in the central area of the data. Also, a representation of the measuring ranges has been included in the figure. In that ranges, we would consider that the biosensor provides a reliable measurement. The lower limits of these intervals are the limit of detection or quantification, while the upper limit would be at the maximum concentration we used to perform linear regression. The justification of the upper limit is for the convenience of not extrapolating outside the limits of the data used to perform the regression. In any case, beyond purely mathematical considerations, the upper limit of the measurement interval would be determined by the unacceptable change in the value of uncertainty or sensitivity [6].

Table 2 shows numerical values of the parameters used to build Figure 6. We consider that at least, the data we just listed should be included in some way when we report the performance of a new analytical system or biosensor detecting a particular molecule or bioanalyte.

**Figure 7:**
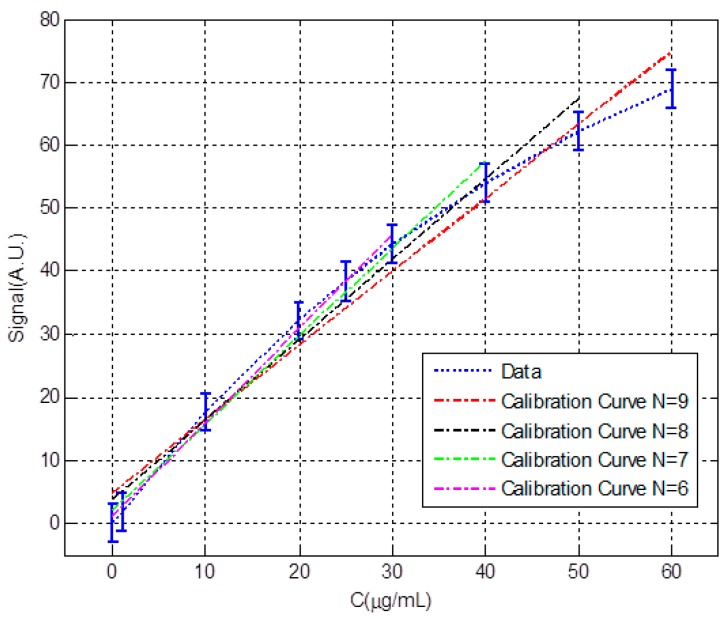
Data and calibration lines for different measuring intervals for the given example.

Figure 7 shows a detail of Figure 5, for the concentration range between 0 and 60 μg/mL, along with four calibration lines constructed with the first 9, 8, 7 and 6 data points. In Table 3 we can see the numerical values associated with each calibration line so that they can be compared. When less data is taken, the slope is higher and the sensitivity increases. The increase in slope causes both the limit and detection and the limit of quantification to decrease. Similarly, the uncertainty values decrease. However, it is important to note that the calibration lines cannot be used to extrapolate out of range, therefore, lines constructed with fewer points, and with a better detection limit, have a narrower measuring range.

**Figure 8:**
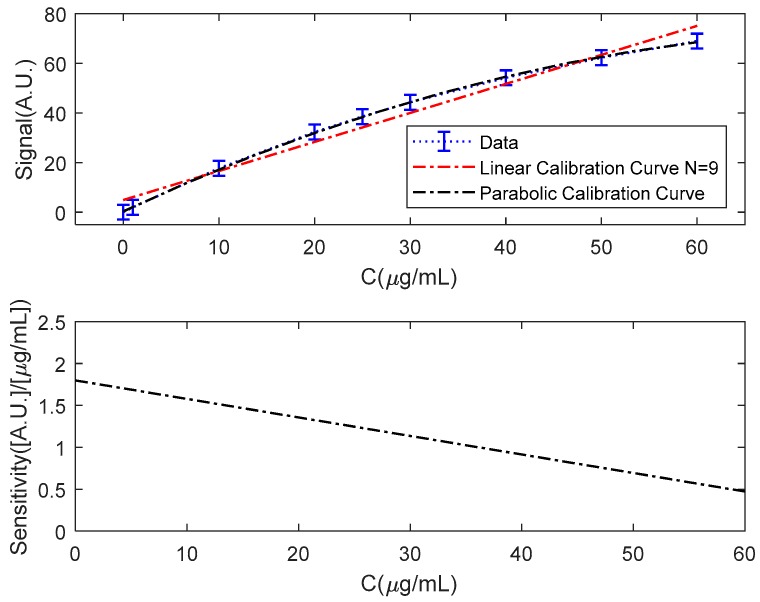
Data, calibration line N = 9 and parabolic fit and parabolic fit sensitivity.

To avoid this problem, higher-order calibration curves can be generated. Depending on the type of sensor and the type of measurement, different mathematical functions can be used to fit the data in the area of interest [27]. Figure 8 shows the first nine data, the linear fit, the parabolic fit given by the Equation (34) and the variation of the sensitivity:

(34) $$\mathrm{Signal}\left(C\right)=-0.0110C^{2}+1.7987C+0.3103$$

**Figure 9:**
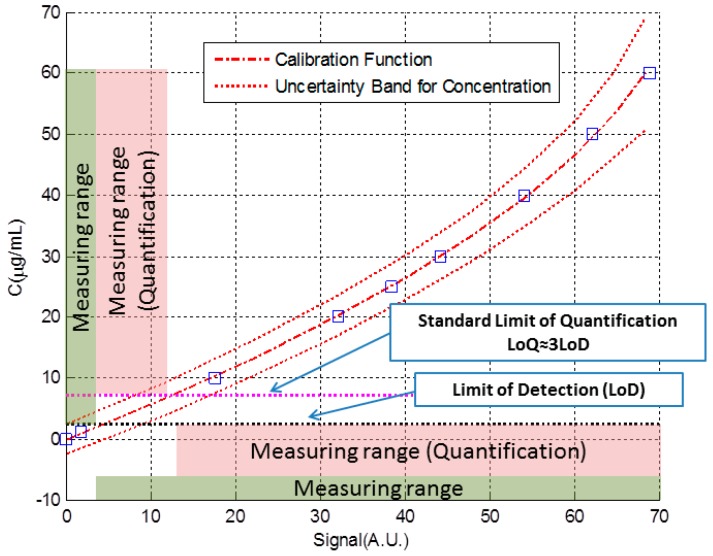
Data, calibration curve for parabolic fit, uncertainty band, limits of detection and quantification and measuring intervals for the given example.

If the parabolic fit is used (Figure 9), the sensitivity decreases with the concentration, being maxima for the null concentration. Figure 9 shows the variation of the sensitivity for the case discussed; and the uncertainty band and the limits of detection and quantification. Using the maximum slope of the parabolic adjustment and neglecting the contribution to the uncertainty of the adjustment process we can determine the limit of detection by Equation (35):

(35) $$\;C_{LoD}=k\left[\frac{1}{a}\sqrt{\frac{s_{B}^{2}}{n}+\frac{R^{2}}{12}}\right]$$

In this case, we obtain a lower detection limit of 2.4 μg/mL for a maximum sensitivity of 1.8 A.U/(μg/mL). Assuming that the uncertainty in the measurement of the concentration is of the order of the detection limit and inversely proportional to the sensitivity, it should increase for higher concentrations. Obviously, for low sensitivities, the same uncertainty in the signal leads to a greater change in the value of the measured concentration.

### 3.2. Experimental Immunoassay

Table 4 shows calibration data of a biochip composed by a set of n=6 BICELLs (biophotonics sensing cells) [28], to be used as antibody-based (anti-IgG) sensors label-free biosensor. The calibration is performed over N=11 calibration points from $C_{1}=1$ μg/mL to $C_{11}=$ 100 μg/mL. Uncertainties of concentrations $C_{i}$ are negligible against other uncertainty sources. The transduction signal $y_{ij}$ is the resonant dip shift observed when the sensor is exposed to antibody concentrations $C_{i}$. The readout instrument resolution is R = 0.12 nm. Values $y_{ij}$ represent the mean of seven repeated measurement over the same concentration $C_{i}$ using the cell number *j*. Columns on the right are the mean ${\bar{y}}_{i}=\sum_{j=1}^{n}y_{ij}/n$ and the standard deviation $s_{i}={\left[\sum_{j=1}^{n}{\left(y_{ij}-{\bar{y}}_{i}\right)}^{2}/\left(n-1\right)\right]}^{1/2}$. The objective of the calibration is to estimate a common calibration curve that could be used with any of the six cells. Therefore, the lack of reproducibility [8] among cells should be taken into account when estimating uncertainties. Please note that $s_{i}$ estimates reproducibility among cells and not repeatability [8,11].

After representing graphically data of Table 4 two facts can be easily observed:

○ The biosensor response is non-linear, with a noticeable lack of repeatability at the end of its scale (over 20 μg/mL).
○ Variability increases with concentration probably due to differences in sensing cells or biofuntionalization performance among other possible factors (standard deviations $s_{i}$ do not pass the Hartley’s test [29]).

In order to estimate the increase of the variability with concentration we have used the procedure described in [10] (part II, Section 5.3.2) which assumes a linear dependence of $s_{y}$ with C. Finally, we have obtained the following result (for the range from 1 μg/mL to 20 μg/mL):

(36) $${\hat{s}}_{y}={\hat{s}}_{y}\left(C\right)=\left(0.049+0.0126\cdot C\;\left[\mu g/\mathrm{mL}\right]\right)\;\mathrm{nm}$$

${\hat{s}}_{y}={\hat{s}}_{y}\left(C\right)$ is an estimation of $s_{y}$ as a linear function of C. Now, if we compute the normalized standard deviations ${\tilde{s}}_{i}=s_{i}/{\hat{s}}_{y_{i}}$, where ${\hat{s}}_{y_{i}}={\hat{s}}_{y}\left(C_{i}\right)$, then the Hartley’s test is passed for ${\tilde{s}}_{i}$. Please note that ${\hat{s}}_{y}={\hat{s}}_{y}\left(C\right)$ estimates the reproducibility between cells, not the repeatability of a certain cell. This is the key point that permits using a common calibration curve for the complete set of six cells.

We have performed weighted least squares fitting (weights proportional to $1/{\hat{s}}_{y_{i}}^{2}$) using polynomials from degree g=1 to g=4 and a five parameter logistic function (5PL). For each fit, we have calculated the following parameters which have been collected in Table 5:

○ The degrees of freedom of the problem =N−k , where k is the number of the parameters to be determined during the fitting: five for the 5PL, g+1 for the polynomials.
○ The weighted sum of squares $=\sum_{j=1}^{N}{\left(y_{ij}-{\hat{y}}_{i}\right)}^{2}/{\hat{s}}_{y_{i}}^{2}$, where ${\hat{y}}_{i}$ is ${\hat{y}}_{i}=f\left(C_{i}\right)$. f(C) is the fitted calibration curve.
○ The critical value $\chi_{c}^{2}$ of a Chi-square distribution with ν=N−k degrees of freedom corresponding to a confidence level of β=95%.
○ The Akaike Information Criterion (corrected) [27,30]:
  AICc=N·log(Q/N)+2k+2k(k+1)/(N−k−1).

To select the calibration curve we have follow the procedure described in [27], based on the use of the Akaike Information Criterion (AICc). The lower is the AICc the better is the model adequacy. We have used AICc and not the Bayesian Information Criterion (BIC) because according to [30] (Section 6.4.3) AIC-type criteria give better results in areas such as medicine and biology. The minimum AICc value corresponds to a second degree polynomial (parabolic fit). Please note that the parabolic fit passes the Chi-squared test. Probably, the reason why the parabolic fit is the better alternative is related with the low number of calibration points retained (only seven). With a low number of calibration points, curves with many parameters (as fourth degree polynomial or a five parameter logistic function) cannot be fitted with confidence. When a logistic function (5PL) have to be fitted, e.g., when information about dynamic signal range or working range of the biosensor is needed, more calibration points should be used, at least nine, to retain enough information and these calibration points should almost reach the saturation of the biosensor. Otherwise the goodness of the fit cannot be guaranteed.

**Figure 10:**
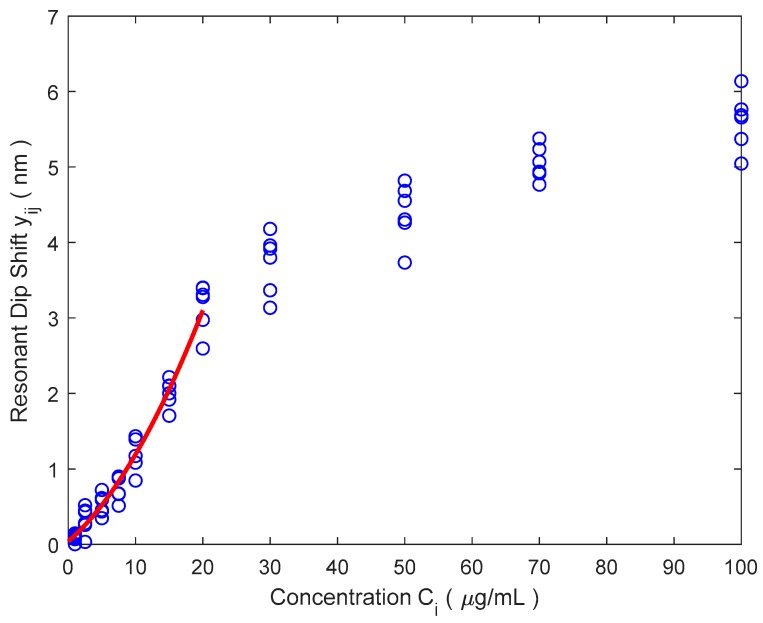
Data from Table 4 and calibration curve for parabolic fit.

Figure 10 shows the calibration data of Table 4 with the selected calibration curve (parabolic fit, g=2). The numerical results corresponding to this calibration curve are the following and have been calculated following a similar procedure to that described in [31]:

(37) $$y=\mathrm{Signal}\left(C\right)=f\left(C\right)=x_{1}+x_{2}\cdot C+x_{3}\cdot C^{2}$$

$$x_{1}\;=\;0.040\;\mathrm{nm}\;u\left(x_{1}\right)\;=\;0.031\;\mathrm{nm}\;r\left(x_{1},x_{2}\right)=-0.80$$

$$x_{2}\;=\;0.078\;\mathrm{nm}/(\mu g/\mathrm{mL})\;u\left(x_{2}\right)\;=\;0.012\;\mathrm{nm}/(\mu g/\mathrm{mL})\;r\left(x_{1},x_{3}\right)=+0.67$$

$$x_{3}\;=\;0.00378\;\mathrm{nm}/(\mu g/\mathrm{mL}{)}^{2}\;u\left(x_{3}\right)\;=\;0.00071\;\mathrm{nm}/(\mu g/\mathrm{mL}{)}^{2}\;r\left(x_{2},x_{3}\right)=-0.94$$

The biosensor sensitivity a is now the derivative of the calibration function f(C):

(38) $$\;a\left(C\right)=x_{2}+2x_{3}C$$

The sensitivity varies linearly from 0.078 nm/μg/mL) at C = 0 to 0.229 nm/(μg/mL) at C = 20 μg/mL and the readout instrument resolution R = 0.12 nm is constant. The quotient between instrument resolution and the sensitivity (R/a), that could be defined as the resolution of the biosensing system, decreases from 1.5 μg/mL at C = 0 to 0.52 μg/mL at C = 20 μg/mL due to the variation of the sensitivity.

After the six cells of the biochip have been calibrated, a common calibration curve (37) can be used, with any of the six cells, to make measurements of anti-IgG concentrations. But now, we need to use inverse function of (37). That is:

(39) $$\;C=f^{-1}\left(y\right)=\frac{-x_{2}-\sqrt{x_{2}^{2}-4x_{3}\left(x_{1}-y\right)}}{2x_{3}}$$

Propagating uncertainties in a similar way as has been done before in Section 3.1 we could determine U(C) as a function of the reproducibility ${\hat{s}}_{y}={\hat{s}}_{y}\left(C\right)$, the resolution *R*, the standard uncertainties $u\left(x_{k}\right)$ of the curve parameters $x_{k}$ and their correlation factors $r\left(x_{i},x_{j}\right)$. If necessary, other uncertainty components can be added.

When the sensor is working close to zero concentration, contribution of the second degree term in the calibration curve is negligible. We could write $y=f\left(C\right)≅x_{1}+x_{2}\cdot C$. Therefore, the estimation of the LoD could be done using the same procedure described in Section 2 (Equation (32)) using the following data:

$$b=x_{1}\;=\;0.040\;\mathrm{nm}\;u\left(b\right)=u\left(x_{1}\right)\;=\;0.031\;\mathrm{nm}\;r\left(a,b\right)=r\left(x_{1},x_{2}\right)=-0.80$$

$$a=x_{2}\;=\;0.078\;\mathrm{nm}/(\mu g/\mathrm{mL})\;u\left(a\right)=u\left(x_{2}\right)\;=\;0.012\;\mathrm{nm}/(\mu g/\mathrm{mL})$$

$$s_{B}=\hat{s}\left(C=0\right)\;=\;0.049\;\mathrm{nm}\;R=0.12\;\mathrm{nm}$$

Only one cell is used, so n=1 k=3 (coverage factor)

$$C_{LoD}=\frac{k}{a}\sqrt{\frac{{\hat{s}}^{2}\left(C=0\right)}{1}+\frac{R^{2}}{12}+u^{2}\left(b\right)}=\frac{3\sqrt{{\left(0.049\;nm\right)}^{2}+\frac{{\left(0.12\;nm\right)}^{2}}{12}+{\left(0.031\;nm\right)}^{2}}}{0.078\;\mathrm{nm}/\left(\mu g/\mathrm{mL}\right)}=2.6\;\mu g/\mathrm{mL}$$

**Figure 11:**
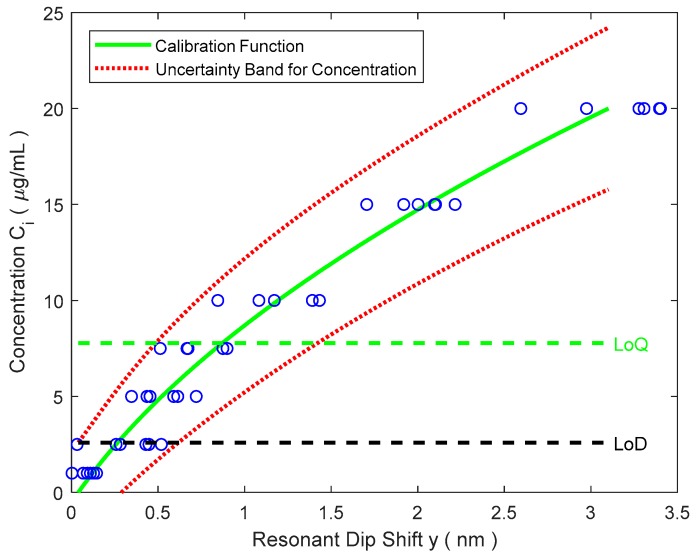
Inverse calibration function and uncertainty bands.

Figure 11 shows the calibration data, the (inverse) calibration function, the uncertainty bands, the limit of detection (LoD) and the limit of quantification (LoQ ≈ 3·LoD). The uncertainty varies on a quasi-linear way from 2.6 μg/mL near zero concentration to 4.2 μg/mL at *C* = 20 μg/mL.

## 4. Conclusions

Analytical systems and biosensors use calibration curves that translate the transduction signal into an analyte concentration. In this article, we have analyzed how should be estimated the limit of detection, limit of quantification, analytical sensitivity and the uncertainty of measurements. Moreover, we have been explained whether the resolution and statistical uncertainty should be considered for the calculation of the limit of detection. However, the performance of a biosensor is not only characterized by the limit of detection. Some additional information should be provided to give us a better idea of their behavior and performance. In this paper, we also propose a method and criteria for supplying the measuring range and uncertainty along the measurement range. With this method, the value of the limit of detection arises naturally from this model as the limit at which uncertainty tends when the concentration approaches to zero.

If we use a line as a function of calibration it is convenient to indicate the range of measurement in which we consider it valid. Its slope is the analytic sensitivity throughout the range. This slope will be used to determine the detection limit. Limits of detection or quantification are the lower limits of the measurement range whereas the upper limit can be defined by the last point used to perform the linear regression due to the convenience of not extrapolating. The typical shape of the immunoassay curves tending to saturation, leading to decrease progressively the slope when the concentration is increased. For this reason, many times the linear regressions have a higher slope with the fewer points close to the null concentration. This makes the detection limits better and the band of uncertainty narrower, but shortens the measuring range.

If better approximations are used to the data, different to a simple linear fitting, the analytic sensitivity stops being constant in the measurement interval. In general, the trend is to decrease with the concentration. In these situations, a relevant problem may usually be the selection of the possible mathematical model that better represents the nonlinear behavior of the data. An alternative could be the use of the Akaike Information Criterion (the corrected version, AICc) as a help during the process of the selection of the model.

## Figures and Tables

**Table 1:** Numerical values represented in Figure 5.

**C (μg/mL)**	0	1	10	20	25	30	40	50	60	100	200	300	400	500
**Signal (A.U.)**	0	1.9	17.7	32.3	38.5	44.2	54.1	62.2	68.9	85.7	97.9	99.0	99.1	99.2
**S (A.U.)**	3	3	3	3	3	3	3	3	3	3	3	3	3	3

**Table 2:** Numerical values of the parameters of Figure 6.

a([A.U.]/[μg/mL]	b([A.U.])	u_a_([A.U.]/[μg/mL]	u_b_([A.U.])	r
1.17	4.88	0.05	1.66	−0.79
LoD (μg/mL)	LoQ (μg/mL)	U_min_ (μg/mL)	U_max_ (μg/mL)	C_Max_(μg/mL)
5.7	16.8	4.5	6.3	60

**Table 3:** Numerical values of calibration lines, uncertainty band, limits of detection and quantification and measuring intervals for the given example.

	N = 9	N = 8	N = 7	N = 6
a([A.U.]/[μg/mL]	1.17	1.27	1.38	1.49
b([A.U.])	4.88	3.37	2.03	1.04
LoD (μg/mL)	5.7	5.4	5.1	4.9
LoQ (μg/mL)	17.1	16.2	15.3	14.7
C_max_(μg/mL)	60	50	40	30
U_min_ (μg/mL)	4.5	4.3	4	3.8
U_max_ (μg/mL)	6.3	5.9	5.6	5.1

**Table 4:** Numerical values from calibration of six BICELLs (Biophotonic Sensing Cells) for anti-IgG detection.

Concentration (μg/mL)	Transduction Signal $y_{\mathrm{ij}}$ (nm)						Mean ${\bar{y}}_{i}$ (nm)	*S_i_* (nm)
	*j* = 1	*j* = 2	*j* = 3	*j* = 4	*j* = 5	*j* = 6		
1	0.13	0.15	0.00	0.09	0.11	0.07	0.09	0.05
2.5	0.52	0.43	0.03	0.28	0.26	0.45	0.33	0.18
5	0.61	0.72	0.44	0.35	0.59	0.45	0.53	0.14
7.5	0.87	0.90	0.67	0.87	0.67	0.51	0.75	0.16
10	1.43	1.39	1.17	1.39	1.08	0.85	1.22	0.23
15	2.22	2.10	2.00	2.10	1.92	1.71	2.01	0.18
20	3.40	3.28	3.31	3.40	2.97	2.60	3.16	0.32
30	3.92	3.80	3.96	4.18	3.37	3.14	3.73	0.40
50	4.55	4.82	4.68	4.30	4.26	3.73	4.39	0.39
70	5.38	5.07	5.24	4.94	4.77	4.91	5.05	0.22
100	6.14	5.69	5.76	5.66	5.37	5.05	5.61	0.37

**Table 5:** Numerical values from calibration of six Biophotonic Sensing Cells (BICELLs) for anti-IgG detection.

Type of Curve	Deegres of Freedom ν	Weighted Sum of Squares Q	Chi-Square Critical Value $\chi_{c}^{2}$	Akaike Information Criterion AICc
Polynomial *g* = 1	5	37.1	11.1	18.7
Polynomial *g* = 2	4	8.66	9.49	15.5
Polynomial *g* = 3	3	6.31	7.81	27.3
Polynomial *g* = 1	2	4.16	5.99	66.4
5PL	2	2.01	5.99	61.3
